# LC/MS-based untargeted lipidomics reveals lipid signatures of nonpuerperal mastitis

**DOI:** 10.1186/s12944-023-01887-z

**Published:** 2023-08-08

**Authors:** Xiaoxiao Chen, Shijun Shao, Xueqing Wu, Jiamei Feng, Wenchao Qu, Qingqian Gao, Jiaye Sun, Hua Wan

**Affiliations:** 1 Department of Breast, Shuguang Hospital, Shanghai University of Traditional Chinese Medicine, Shanghai, 200001 China; 2 Shanghai University of Traditional Chinese Medicine, Shanghai, 200000 China

**Keywords:** Nonpuerperal mastitis, Inflammatory disease, Lipidomics, Triacylglycerol, Arachidonic acid

## Abstract

**Background:**

Nonpuerperal mastitis (NPM) is a disease that presents with redness, swelling, heat, and pain during nonlactation and can often be confused with breast cancer. The etiology of NPM remains elusive; however, emerging clinical evidence suggests a potential involvement of lipid metabolism.

**Method:**

Liquid chromatography‒mass spectrometry (LC/MS)-based untargeted lipidomics analysis combined with multivariate statistics was performed to investigate the NPM lipid change in breast tissue. Twenty patients with NPM and 10 controls were enrolled in this study.

**Results:**

The results revealed significant differences in lipidomics profiles, and a total of 16 subclasses with 14,012 different lipids were identified in positive and negative ion modes. Among these lipids, triglycerides (TGs), phosphatidylethanolamines (PEs) and cardiolipins (CLs) were the top three lipid components between the NPM and control groups. Subsequently, a total of 35 lipids were subjected to screening as potential biomarkers, and the chosen lipid biomarkers exhibited enhanced discriminatory capability between the two groups. Furthermore, pathway analysis elucidated that the aforementioned alterations in lipids were primarily associated with the arachidonic acid metabolic pathway. The correlation between distinct lipid populations and clinical phenotypes was assessed through weighted gene coexpression network analysis (WGCNA).

**Conclusions:**

This study demonstrates that untargeted lipidomics assays conducted on breast tissue samples from patients with NPM exhibit noteworthy alterations in lipidomes. The findings of this study highlight the substantial involvement of arachidonic acid metabolism in lipid metabolism within the context of NPM. Consequently, this study offers valuable insights that can contribute to a more comprehensive comprehension of NPM in subsequent investigations.

**Trial registration:**

Shuguang Hospital Affiliated to Shanghai University of Traditional Chinese Medicine (Number: 2019-702-57; Date: July 2019).

**Supplementary Information:**

The online version contains supplementary material available at 10.1186/s12944-023-01887-z.

## Background

NPM is a chronic inflammatory breast disease characterized by diverse clinical presentations [1–3]. These manifestations encompass an inverted nipple, discharge, lumps, or ruptures, which may exhibit recurrence [4, 5]. Severe cases of NPM can result in both aesthetic breast alterations and mental and physical distress for affected individuals [6]. Furthermore, imaging findings can easily lead to confusion between NPM and breast cancer [7–9]. Therefore, understanding NPM mechanisms as well as lipid biomarkers will facilitate precise diagnosis and treatment.

The current understanding acknowledges that the development of NPM may arise from the heightened permeability of the mammary ducts caused by physical or chemical stimuli such as the presence of lobular mesenchymal infiltrates and luminal secretions (e.g., retained milk). Consequently, this triggers inflammation in the mesenchymal tissue, thereby prompting the infiltration of immunocompetent cells and the subsequent formation of a delayed hypersensitivity reaction [3, 10, 11]. Our clinical observation has identified a notable presence of lipid-like secretions in the breasts of certain patients with NPM. However, a dearth of literature has explored the association between NPM and lipid metabolism. One retrospective study found that individuals with NPM had significantly lower levels of HDL but elevated lipoprotein compared to a control group with benign masses [12]. A separate study showed that 54% of a cohort of 90 patients with NPM were classified as obese (BMI > 30 kg/m²) [13]. In light of these findings, we propose that NPM has abnormal lipid metabolism, which may play an important role in NPM. The application of lipidomics analysis holds the potential to enhance comprehension of NPM by discerning metabolic disruptions within the breast.

Lipids are indispensable metabolites that play pivotal roles in various cellular functions and can serve as a direct indicator of cellular metabolic status [14]. Recent advancements in lipidomics analysis have facilitated the identification and quantification of lipid species in both healthy and diseased states [15], thereby enabling the discovery of disease biomarkers.

The lipidomics data were acquired by using LC/MS. Our study aimed to characterize the metabolic dysregulation associated with NPM and identify its lipid profile. By considering these lipidomics findings, we anticipate enhancing our comprehension of the modifications occurring in lipid molecules within the organism, thereby providing insights into the initiation of NPM.

## Methods

### Reagents and chemicals

Isopropanol (mass spectrometry grade) and acetonitrile (mass spectrometry grade) were purchased from Thermo Fisher Company (Waltham, USA); the Water 2777 C UPLC instrument and Water Xevo G2-XS QTOF mass spectrometer were purchased from Waters Company (Wilmslow, UK).

### Participants

This study was approved by the Medical Research Ethics Committee of Shuguang Hospital Affiliated to Shanghai University of Traditional Chinese Medicine. The diagnosis of NPM was made according to clinical manifestations, radiological images and pathology. The typical pathological feature under the microscope is the formation of noncaseating granulomas centered on breast lobules. In the center of the granuloma, microabscesses dominated by neutrophil infiltration surrounded by histiocytes, macrophages, and multinucleated giant cells can be seen. The outermost periphery is surrounded by lymphocytes, plasma cells and other inflammatory cells and fibroblasts [1]. Prior to their participation in the study, all individuals provided informed consent. The study encompassed a cohort of 20 patients diagnosed with NPM, alongside a control group consisting of 10 patients with fibroadenoma.

### Breast tissue collection

In the case of NPM, the excision involved the removal of diseased breast tissue, whereas for fibroadenoma cases, the excision encompassed the removal of the surrounding normal breast tissue adjacent to the lesion. Throughout the surgical procedure, adherence to sterility principles was imperative. We collected approximately 200 mg of breast tissue, immediately placed it in liquid nitrogen for at least 15 min and froze it at -80 °C for lipidomics analysis.

### Lipid extraction

Approximately 25 mg of the sample was incubated with 800 µL of extract solution (dichloromethane:methanol = 3:1) [16]. They were then homogenized using a tissue lyser and kept in a refrigerator at -20 °C for 2 h. Subsequently, the samples were centrifuged twice at 25,000 × g for 15 min each at 4 °C. The supernatant obtained (600 µL) was freeze-dried and reconstituted with a lipid complex solution (isopropanol: acetonitrile: water = 2:1:1). The resulting mixture was centrifuged for 20 min at 25,000 g and 4 °C. The supernatant was then collected and transferred to a 96-well microplate for LC‒MS analysis.

Quality control (QC) samples were prepared at the same time (mixing the prepared experimental samples), and the extracted samples were tested on the machine. A QC sample was used to balance the instrument (monitoring the state of the instrument during liquid chromatography‒mass spectrometry), and then a QC sample was interspersed per 10 test samples. The last three QC samples ended the experiment.

### Untargeted lipidomics analysis

First, all chromatographic separations were performed using an ultra-performance liquid chromatography (UPLC) system (Waters, Wilmslow, UK). An ACQUITY UPLC CSH C18 column (100 mm×2.1 mm, 1.7 μm, Waters, Wilmslow, UK) was used for separation. The column oven was maintained at 55 °C. The flow rate was 0.4 ml/min, and the mobile phase consisted of solvent A (ACN:H2O = 60:40, 0.1% formic acid and 10 mM ammonium formate) and solvent B (IPA:ACN = 90:10, 0.1% formic acid and 10 mM ammonium formate). Gradient elution conditions were set as follows: 0 ~ 2 min, 40–43% phase B; 2 ~ 7 min, 50–54% phase B; 7.1 ~ 13 min, 70–99% phase B; 13.1 ~ 15 min, 40% phase B. The injection volume for each sample was 5 µL.

A high-resolution tandem mass spectrometer Xevo G2 XS QTOF (Waters, Wilmslow, UK) was used to detect metabolites eluted from the column. The Q-TOF was operated in both positive and negative ion modes. For positive ion mode, the capillary and sampling cone voltages were set at 3.0 kV and 40.0 V, respectively. For negative ion mode, the capillary and sampling cone voltages were set at 2 kV and 40 V, respectively. The mass spectrometry data were acquired in Centroid MSE mode. The TOF mass range was from 100 to 2000 Da in positive mode and 50 to 2000 Da in negative mode. The survey scan time was 0.2 s. For MS/MS detection, all precursors were fragmented using 19–45 eV, and the scan time was 0.2 s.

### Statistical analyses

By importing the original detection data into Progenesis QI 2.2 software (Waters, USA) for peak extraction, the mass-to-charge ratio, retention time, and ion area information related to the metabolites can be obtained. Data preprocessing was performed using metaX software, and the steps included filtering out low-quality ions (first removing ions in the QC sample that contained over 50% missing values, then removing ions in actual samples that contained over 80% missing values); using a K-nearest neighbor method for filling the missing values; using the probabilistic quotient normalization method for data normalization; using the QC-RSC (quality control-based robust LOESS signal correction) method for batch effect correction; filling missing values again; filtering out ions in all QC samples with RSD > 30% (the ions with RSD > 30% fluctuated greatly in the experiment and were not included in the downstream statistical analysis).

Univariate and multivariate analyses were performed to obtain metabolites that differed between groups; the method included parameter tests and nonparametric tests, differential expression multiple analysis, principal component analysis (PCA) and partial least square discriminant analysis (PLS-DA). PCA transforms multiple variables into a few important variables (principal components) by dimensionality reduction techniques. PLS-DA is widely used by the metabolome; among them, PLS, also known as latent projection, is a linear regression method. Both a supervised method (PLS-DA) and an unsupervised method (PCA) were carried out to discriminate the differences in lipid profiles between the two groups. To screen for differential metabolites, VIP > 1 and *p*<0.05 were used, and p<0.05 was typically fixed. Finally, the lipids were identified and analyzed based on the database HMDB. Metabolites were identified in Progenesis QI with retention time, accurate molecular mass, and MS^E^ data. The molecular formula was obtained by searching the HMDB. The metabolite identification was comprehensively scored, considering the database mass matching accuracy of the MS1 identification (the higher the matching accuracy is, the higher the score is), the isotope peak similarity score (the similarity of the isotope distribution between the measured metabolite and the theoretical isotope distribution, the higher the similarity is, the higher the score is) and MS2 matching similarity score. The pipeline of the MS2 matching similarity score is as follows: generate the theoretical fragment spectrum of identified metabolites and compare the experimental MS2 ion spectrum after deconvolution to obtain the similarity score. The figures were generated using MetaboAnalyst version 5.0 (https://www.metaboanalyst.ca/) and in-house R scripts.

Furthermore, this study built a weighted metabolite coexpression network that used the R package “WGCNA”. We used 1–TO (dissTOM) as a distance measure to perform stratified cluster lipid features. The Pearson correlation coefficient between the characteristic genes of each module and each clinical trait was calculated to identify significant clinical modules (*P* < 0.05).

### Workflow

**Fig. 1:**
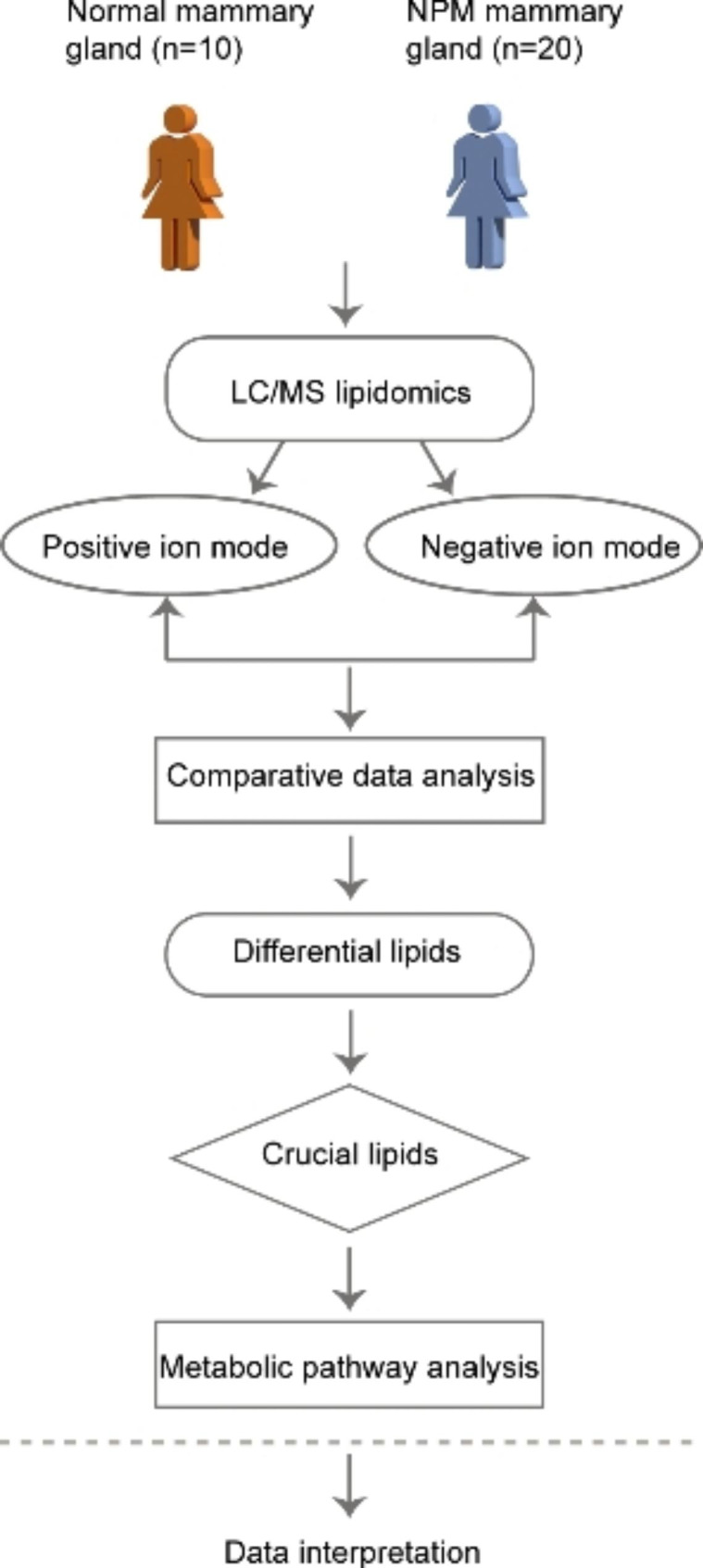
Workflow of this study

The workflow is summarized in Fig. 1.

## Results

### Clinical characteristics of NPM and control

The clinical characteristics of patients with NPM and controls are summarized in Table 1. All participants were enrolled between August 2019 and November 2019. The patients were all females, including 10 patients with fibroadenoma and 20 patients with NPM, and all were diagnosed pathologically. Within the normal control group, only 7 patients had complete lipid information, while age and body mass index (BMI) information was available for all patients. In the NPM group, all 20 cases were completed. The results showed that there were no significant differences in age, serum total cholesterol (TC), TG or low-density lipoprotein-cholesterol (LDL) between the two groups. The BMI and high-density lipoprotein-cholesterol (HDL) of the NPM group were higher and lower, respectively, than those of the control group (*P* < 0.05).

**Table 1:** Clinical Characteristics of the NPM for Lipidomics Analysis

Variables	Control (n = 10)	NPM (n = 20)	*P* value
Age (year)	33.50 ± 8.26	32.40 ± 3.99	0.624
BMI (kg/m^2^)	20.77 ± 2.49	23.93 ± 3.48	0.016
TC (mmol/L)	4.81 ± 0.79	4.38 ± 0.85	0.085
TG (mmol/L)	1.03 ± 0.52	1.65 ± 0.79	0.283
HDL (mmol/L)	1.45 ± 0.38	1.08 ± 0.21	0.005
LDL (mmol/L)	2.83 ± 0.81	2.68 ± 0.73	0.669

Data are presented as the mean ± SD

### Multivariate statistical analysis of lipids in breast tissue

**Fig. 2:**
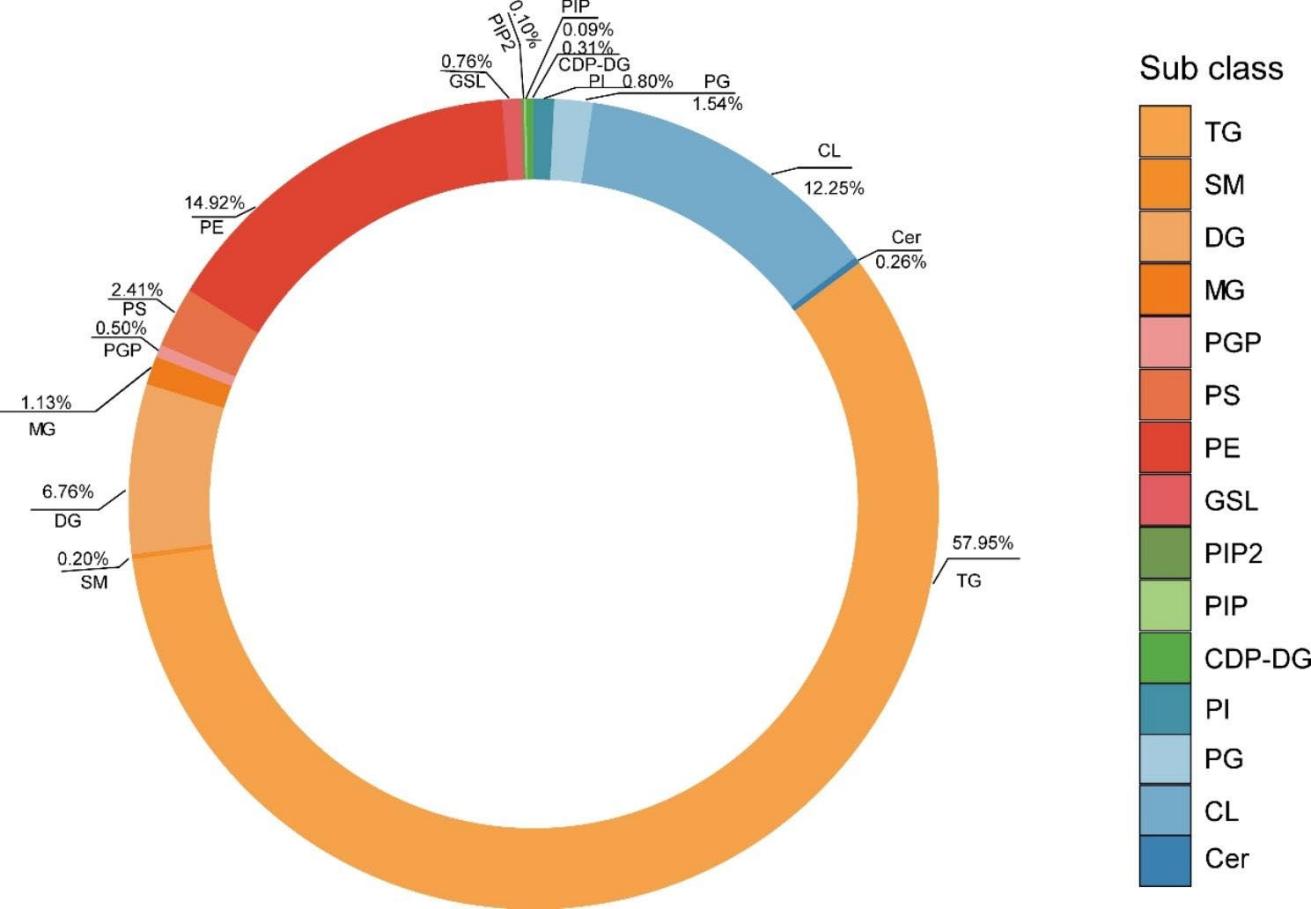
Pie chart of subclass distribution of lipids in breast

A variety of Internet-based databases, for instance, the Human Metabolome Database (HMDB) and lipidmaps, provide access to identify lipids. In total, 14,012 lipids were identified, including 16 subclasses, with the top 5 being TGs (56.21%), PEs (14.47%), CLs (11.89%), diradylglycerols (DGs) (6.56%), and phosphatidylserine (PS) (2.34%) (Fig. 2).

**Fig. 3:**
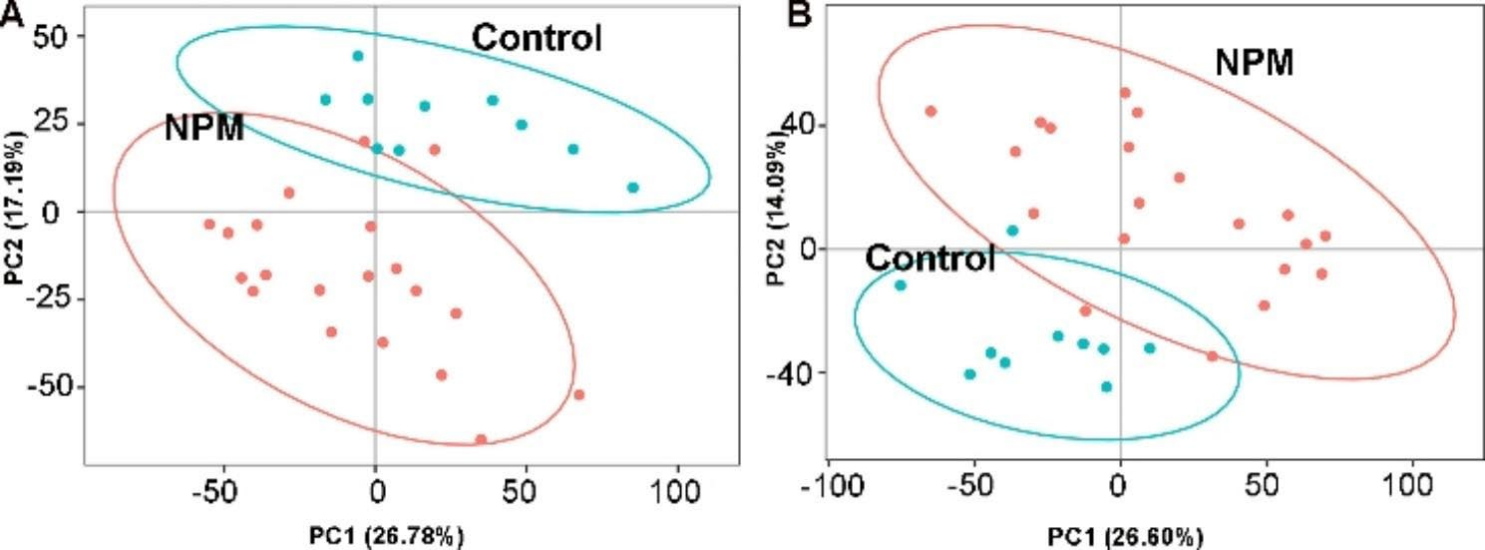
PCA score plots of the NPM and control groups. (**A**) Negative mode; (**B**) Positive mode

**Fig. 4:**
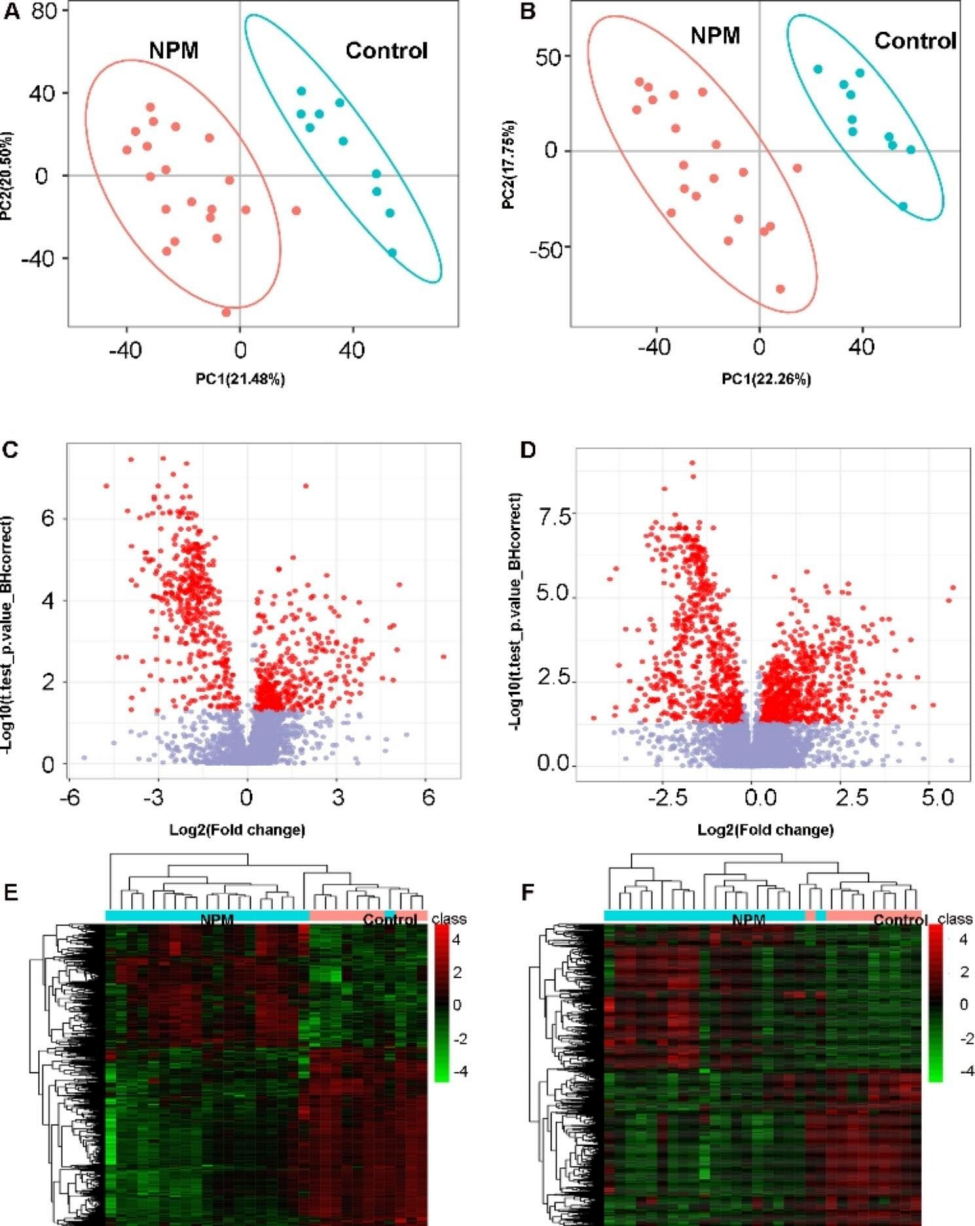
Multivariate statistical analysis for lipid profile between the NPM and control groups. (**A**)(**B**) PLS-DA Discriminant Analysis Model Score Map of two groups in negative and positive modes, respectively. (**C**)(**D**) Volcano plot of two groups in negative and positive modes, respectively. (**E**)(**F**) Differential ion cluster analysis graph of the two groups in negative and positive modes, respectively. The rows in the figure represent differential ions, and the columns represent samples. Colors range from green to red, indicating strength from low to high

To evaluate significant changes in lipids in NPM compared with the control group, first, PCA was conducted to obtain an overview of the difference in the lipidome among the two studied groups (Fig. 3). As shown by the PCA score plot, the NPM group had a distinct lipid metabolic profile from the control group. Then, we performed PLS-DA with two predictive components. The PLS-DA plot showed an *R*^*2*^ of 97.1% and Q^2^ of 72.7% and an R2 of 96.8% and Q2 of 84.4% for negative and positive ions, respectively (Fig. 4A, B). Additionally, a volcano plot and heatmap of changed metabolites between the two groups were generated. A total of 1181 variables were selected in the positive-ion mode and 705 variables in the negative-ion mode. Those points whose fold change was less than or equal to 0.8333 or greater than or equal to 1.2 and whose q-value was less than 0.05 were marked in red, and the rest were marked in blue (Fig. 4C, D). The above results, including the heatmap (Fig. 4E, F), clearly demonstrated that these lipid changes can be clearly distinguished between the two groups.

### Validation of the crucial lipids discriminating NPM from Control

**Fig. 5:**
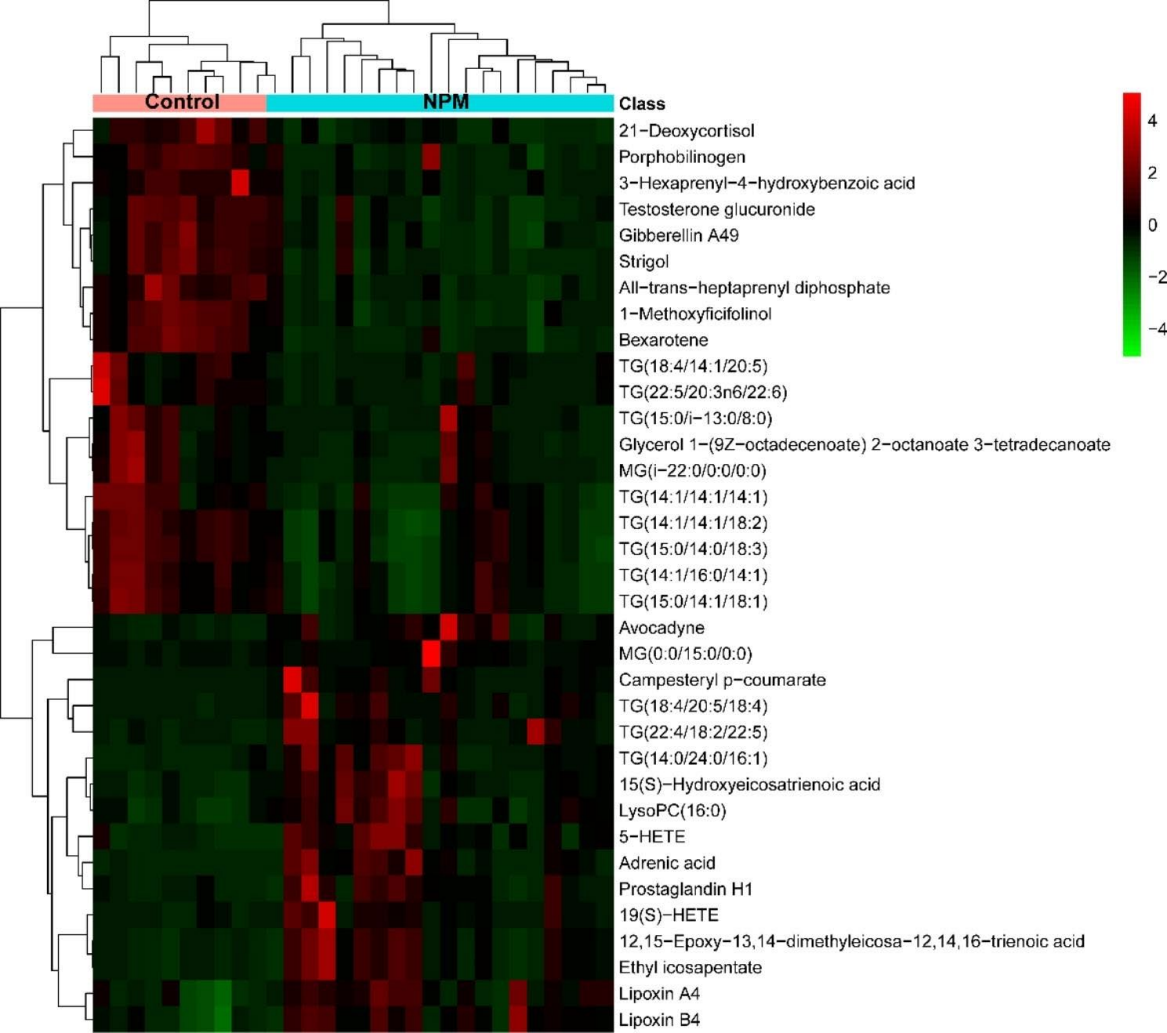
Heatmap of the 35 identified lipids. Different samples (shown as columns) with increased or decreased levels of metabolites (shown as rows) are indicated by red or green

**Fig. 6:**
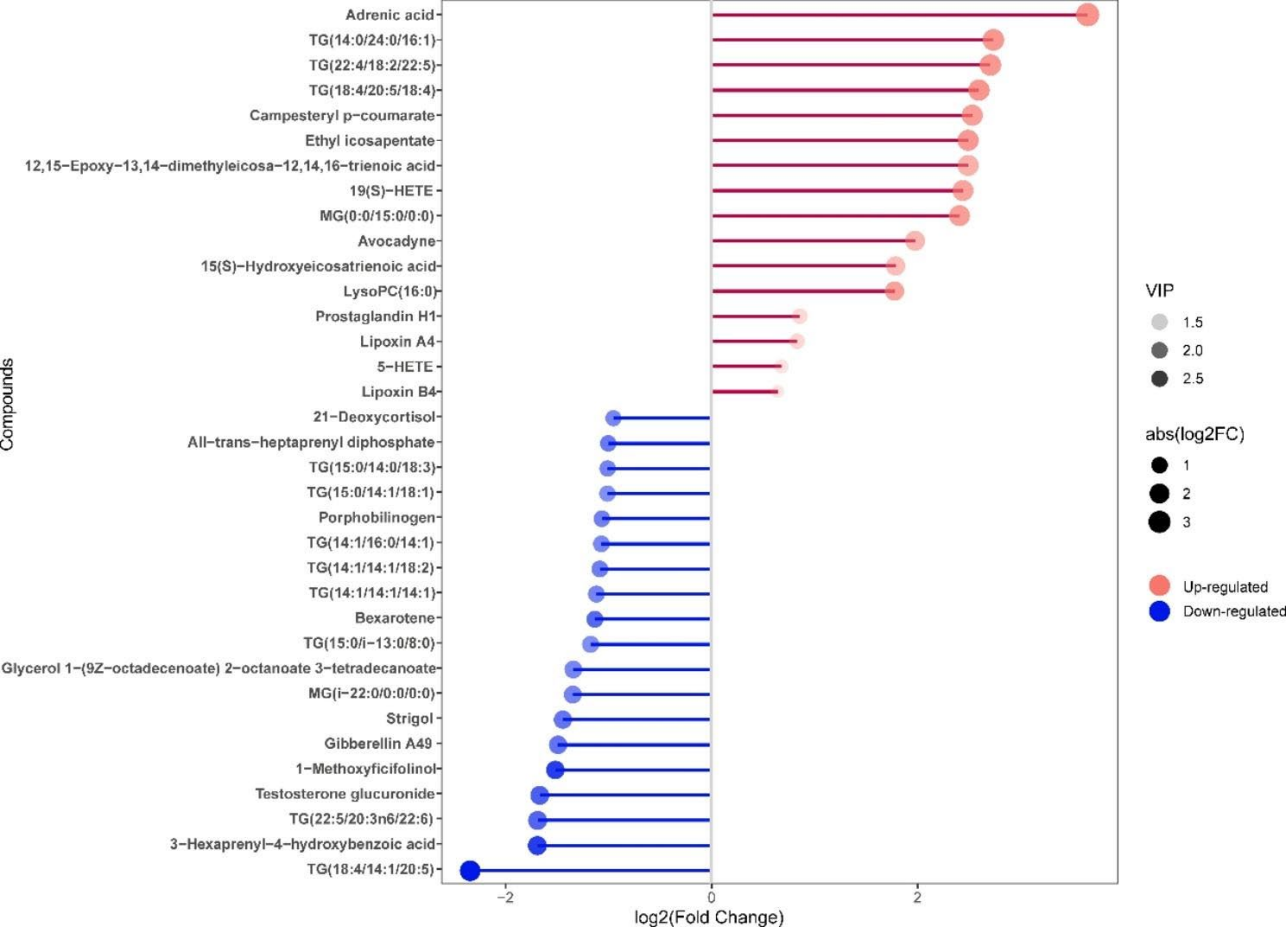
Matchsticks of the 35 markedly upregulated and downregulated lipids. VIP: variable importance in projection

Based on the analysis of metabolic pathways and VIP values, we screened lipids with notable differences as potential biomarkers. Subsequent heatmap analysis revealed 35 lipids that distinguished the NPM from the control (Fig. 5). The lipid species identified included 11 fatty acyl species, 1 isoflavonoid species, 3 steroids and steroid derivative species, 4 prenol lipid species, 1 organonitrogen compound species, 1 glycerophospholipid species, and 14 glycerolipid species. Again, these results confirmed that NPMs undergo significant changes in lipids in comparison with the control. Figure 5 shows the patterns of lipid expression in the two groups based on data analysis through hierarchical clustering and heatmapping. Of the 35 crucial lipids discriminating NPM from the control, 16 were more abundant and 19 were less abundant. A lollipop plot illustrated the above changes (Fig. 6). Among them, the top 5 species that exhibited more abundant lipids were adrenic acid, TG(14:0/24:0/16:1), TG(22:4/18:2/22:5), TG(18:4/20:5/18:4), and campesteryl p-coumarate (Fig. 6).

### Lipid coexpression network modules closely correlated with NPM

**Fig. 7:**
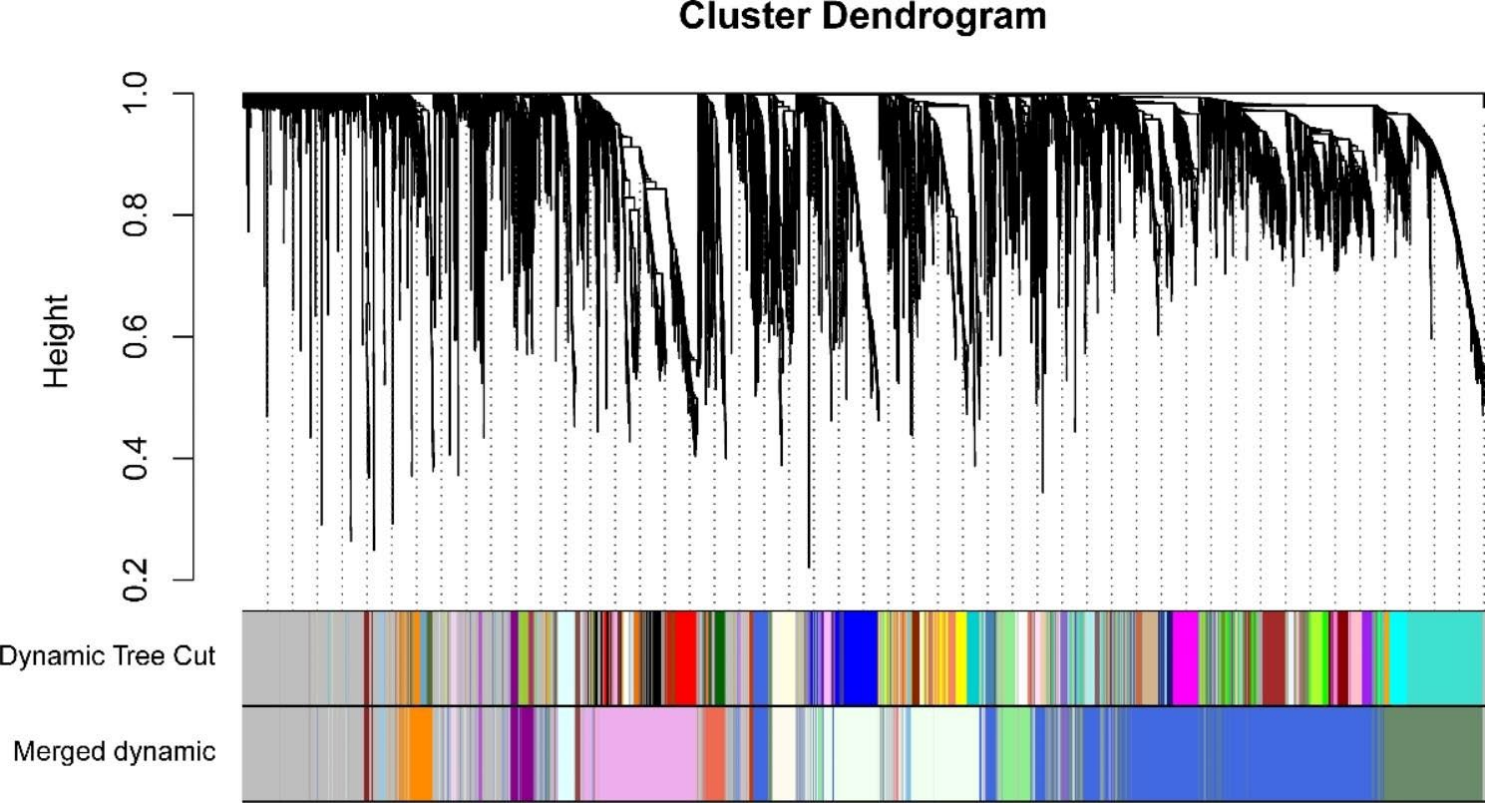
Module cluster trees were used to visualize lipid distributions

**Fig. 8:**
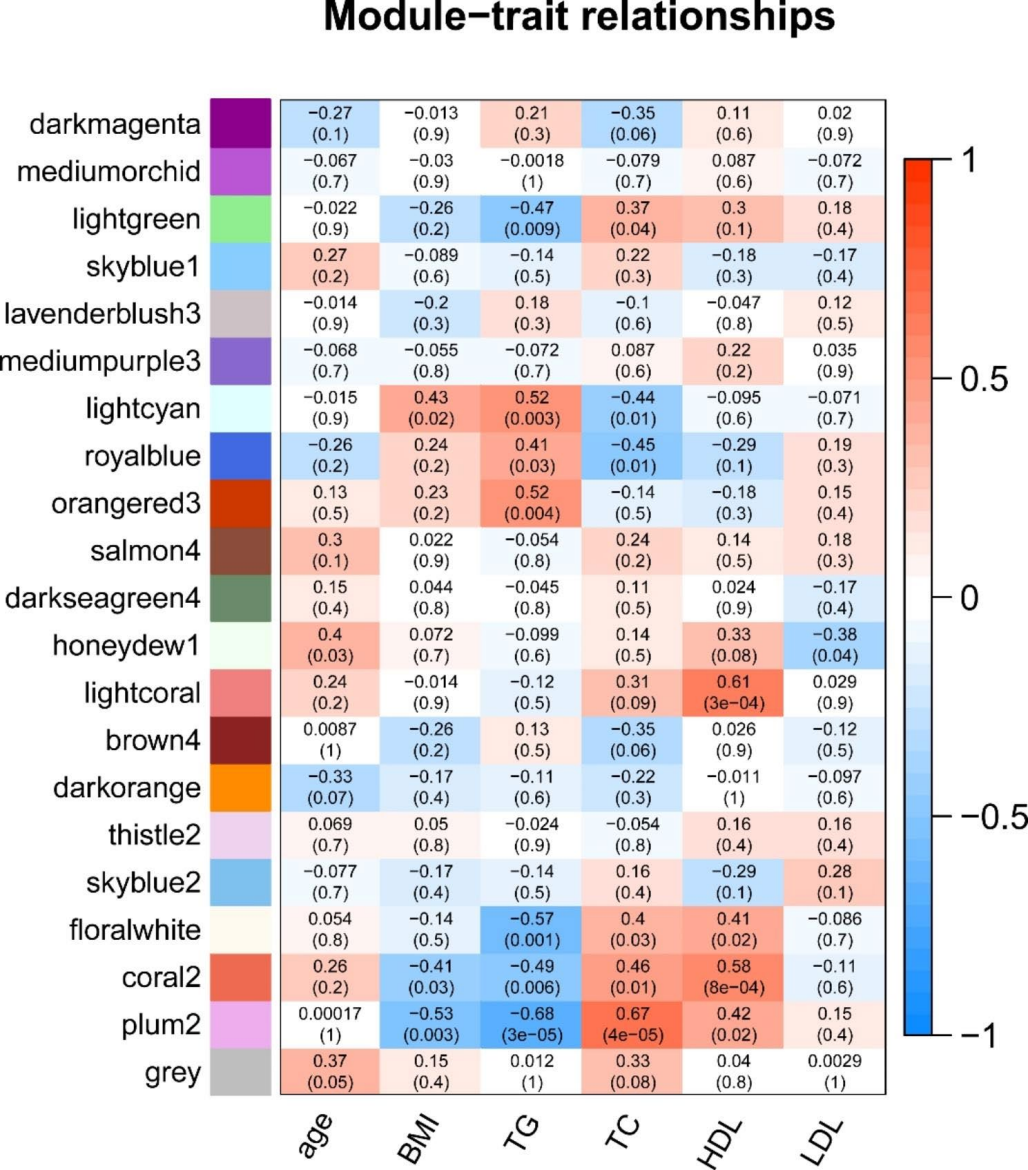
Pearson correlation analysis of the network modules and demographic characteristics of the NPM. Red indicates a positive correlation, blue indicates a negative correlation, and numeric magnitude indicates a correlation. The numbers in the boxes indicate the correlation coefficient, and the numbers in parentheses are *P* values

To explore the correlation between lipids and different clinical indicators, we performed a WGCNA. A hierarchical clustering tree was obtained using hierarchical clustering for dissTOM (Fig. 7), and a total of 21 modules were obtained. A Pearson correlation analysis was conducted to examine the relationships between the network modules and different sample characteristics. The analysis showed a notable positive correlation between HDL and the lightcoral module (R = 0.61, P = 3e-04), while the lightcyan (R = 0.43, P = 0.02) modules showed a positive correlation with BMI, as illustrated in Fig. 8.

### Metabolic pathway

**Fig. 9:**
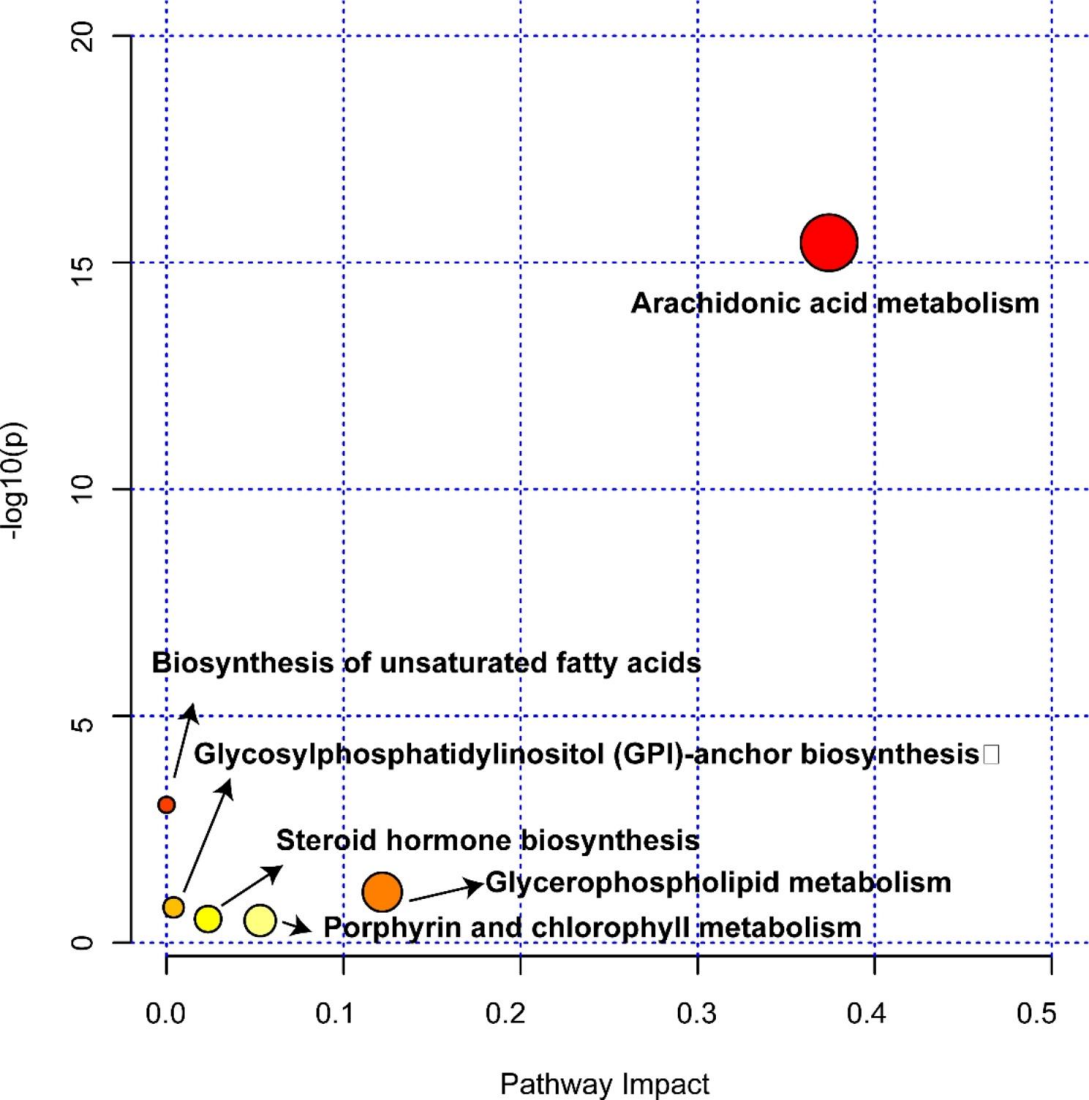
Pathway analysis based on lipids. Circle color and circle size represent the *P* value (y-axis) and pathway impact (x-axis)

Through the utilization of pathway analysis, we conducted an exploration into the potential mechanisms underlying the observed variations in lipids within NPM. These mechanisms encompass arachidonic acid metabolism, glycerophospholipid metabolism, porphyrin and chlorophyll metabolism, glycosylphosphatidylinositol (GPI)-anchor biosynthesis, biosynthesis of unsaturated fatty acids, and steroid hormone biosynthesis (Fig. 9). In addition, specific information, including *P* values, impact factor values, and proportion of altered lipids of each pathway, is shown in Table S2. Notably, arachidonic acid metabolism emerged as the pathway exhibiting the most significant alterations between the NPM and control groups.

## Discussion

As of yet, NPM has not been fully elucidated as to its cause. Even though NPM is benign and self-limiting, it is increasingly valued. On the one hand, the differentiation between NPM and breast cancer is still an urgent problem to be solved; on the other hand, it can cause extensive damage to the breast [17, 18]. The most common causes are autoimmune conditions, lipophilic infections caused by Corynebacterium, and high prolactin levels [19–21]. Additionally, several studies have shown that lipid metabolism may exert a notable influence on NPM [12, 22]. Currently, there is a dearth of definitive evidence concerning the correlation between BMI and NPM. Through an examination of the BMI of both NPM patients and normal controls, investigators discovered that individuals with NPM exhibited higher BMI values [23]. It is noteworthy to mention that within the scope of this study, we observed a potential association between the decrease in HDL and the presence of NPM. However, upon conducting a subsequent regression analysis using a generalized linear equation, it was determined that the abnormality of HDL did not exhibit a statistically significant influence on disease prediction. This outcome could potentially be attributed to the limited sample size utilized in this study, thereby necessitating a larger sample size for future validation. These findings potentially imply aberrant lipid metabolism in NPM patients. Lipidomics, one of the branches of metabolomics, is divided into untargeted lipidomics and targeted lipidomics [24]. Untargeted lipidomics provides a direction for discovering new lipids and metabolic pathways and is a relatively open approach [25]. To the best of our knowledge, an analysis of NPM and control group lipidomics is being conducted for the first time.

LC/MS analysis revealed prominent differences in the lipidome between NPM and the control. Subsequent lipid classification analysis revealed that 85.12% of the lipids were TG, PE, and CL lipids among the different lipid subclasses. TG metabolites showed good diagnostic potential when screened for differential metabolites. TG, including TG(14:0/24:0/16:1), TG(14:1/14:1/14:1), TG(14:1/14:1/18:2), TG(14:1/16:0/14:1), TG(15:0/14:0/18:3), TG(15:0/14:1/18:1), TG(18:4/14:1/20:5), TG(18:4/20:5/18:4), TG(22:4/18:2/22:5), and TG(22:5/20:3n6/22:6), are good lipid biomarker candidates. TGs composed of fatty acids and glycerol are the most abundant lipids in the human body [26]. TG metabolism abnormalities contribute to excessive lipid accumulation and oxidative stress, as well as accelerating inflammatory processes [27, 28]. Glycerophospholipids (GPLs), apart from their role as integral components of cellular membranes, also serve various other functions. As an example, numerous GPLs are involved in signaling, such as producing arachidonic acid, which is the precursor to prostaglandins [29, 30]. Phosphatidylcholine (PC), a type of GPL, can be hydrolyzed to produce LysoPC (LPC). Existing evidence suggests that saturated LPC increases proinflammatory cytokines, exacerbates inflammation and has been implicated in inflammatory diseases [31, 32]. In this study, GPLs constituted a portion of the differential lipid classification, and LysoPC (16:0) was elevated in NPM compared with the control group. Moreover, pathway analysis revealed that NPM may be related to GPL metabolism. Through the analysis of differential lipid pathways, we found that arachidonic acid metabolism was primarily responsible for differential lipid spectrometry measurements. The lipid component of cell membranes, arachidonic acid (AA), is metabolized by three different enzymes: cyclooxygenase (COX), lipoxygenase (LOX), and cytochrome P450 (CYP450) enzymes. AA can be converted into a variety of metabolites that trigger inflammatory responses based on these three metabolic pathways [33]. It is well known that leukotrienes (LTs) derived from AA are potent mediators of inflammation as well as the proliferation of leukocytes [34]. Additionally, both LXA4 and LXB4 have shown promising potential as effective markers in distinguishing between different conditions. The lipoxin family of eicosanoids is commonly synthesized through transcellular lipoxygenase [35]. The production of LXA4 and LXB4 has been observed in various inflammatory conditions [35]. In addition, previous studies have shown that lipotoxins are endogenous lipid modulators that inhibit the aggregation of neutrophils in inflammatory responses and act as a mechanism of host defense [36]. Researchers have also found that LXB4 boosts memory B cells via COX2 [37]. Although many studies [38, 39] have linked NPM to immunity, its precise mechanism of action remains unknown. In summary, screening differential lipids and pathway analysis may provide a new direction for studying the immune-related mechanisms of NPM.

### Study strengths and limitations

To the best of our knowledge, this study represents an inaugural investigation into the lipidomics of breast tissue in patients with NPM. As anticipated, patients with NPM exhibit modified lipid profiles within their breast tissue. As a result of differential identification of lipid species, marker screening and pathway analysis, this paper offers new insights into the understanding of NPM. However, it is important to acknowledge the limitations of this study, namely, the small sample size, necessitating further investigation with larger sample sizes to validate the obtained results. Additionally, it is worth noting that this study employs untargeted lipidomics, which offers a relatively quantitative approach but does not enable the estimation of individual lipid species. Furthermore, lipids exhibit distinct functional characteristics depending on their chain lengths and saturations. A comprehensive understanding of the functional implications resulting from slight variations in carbon number or unsaturation remains incomplete.

## Conclusion

Our study investigated the lipid signature of NPM in breast tissue by LC/MS-based untargeted lipidomics. In terms of lipid category, the alterations in lipid composition observed in breast tissue of NPM patients primarily involve TGs, CLs and PEs, which suggests that NPM is closely related to lipid metabolism disorders and mainly relates to the above three types of lipids. More importantly, we emphasize the importance of arachidonic acid metabolism as a possible driving force in the NPM process. Apart from the significant role of immunity and inflammation in NPM, there is also a possibility that lipid metabolism disorders are involved in disease development. However, further functional and mechanistic studies are needed to shed more light on NPM pathogenesis.

### Electronic supplementary material

Below is the link to the electronic supplementary material.

**Additional file 1**: Fig S1. PCA according to HDL

**Additional file 2**: Supplementary Table S1. Thirty-five differential metabolites between NPM patients and controls

**Additional file 3**: Supplementary Table S2. Exact p values, impact factor values and proportion of altered lipids for each pathway

**Additional file 4**: Fig S2. PCA score plots of the 35 identified lipids in 3D mode

## Data Availability

All data generated or analyzed during this study are available from the corresponding author on reasonable request.

## Abbreviations

TG: Triglyceride
PE: Phosphatidylethanolamines
CL: Cardiolipin
DG: Diglyceride
PS: Phosphatidylserine
FA: Fatty Acyls
PG: Phosphatidylgylcerol
MG: Monoglyceride
PI: Phosphatidylinositol
GSL: Glycosphingolipid
PGP: Phosphatidylgylcerolphosphate
CDP-DG: CDP-glycerol
Cer: Ceramide
SM: Phosphosphingolipid
PIP: Phosphatidylinositol-monophosphates
PIP2: Phosphatidylinositol-bisphosphates LXA4:Lipoxin A4
LXB4: Lipoxin B4
NPM: Non-Puerperal Mastitis
UPLC: Ultra performance liquid chromatography
PCA: Principal component analysis PLS-DA:Partial least square discriminant analysis
TC: Serum total cholesterol
TG: Triglyceride
LDL: Low density lipoprotein-cholesterol
BMI: Body mass index
HDL: High density lipoprotein-cholesterol
AA: Arachidonic acid
COX: Cyclooxygenase
LOX: Lipoxygenase
CYP450: Cytochrome P450
LTs: Leukotrienes
VIP: Variable importance in projection
ROC: Receiver Operating Characteristic
GPL: Glycerophospholipid
WGCNA: Gene coexpression network analysis
