# Supplementary material for: LC/MS-based untargeted lipidomics reveals lipid signatures of nonpuerperal mastitis
Source: Lipids Health Dis. 2023 Aug 8;22:122. doi: 10.1186/s12944-023-01887-z (PMC10408177; doi:10.1186/s12944-023-01887-z)
Supplement: Supplementary file 1 — Additional file 1: Fig S1. PCA according to HDL [file 12944_2023_1887_MOESM1_ESM.docx]

**Table S1 Differential metabolites between NPM patients and controls**

|  | metabolites | | class | Comp-ound ID^a^ | | m.z | RT^b^ | 95%CI^c^ | FDR^d^ | | FC^f^ |
| --- | --- | --- | --- | --- | --- | --- | --- | --- | --- | --- | --- |
| 1 | 12,15-Epoxy-13,14-dimethyleicosa-12,14,16-trienoic acid | | Fatty Acyls | 2.39_329.2475m/z | | 329.247 | 2.385 | 0.635-0.950 | 0.025 | | 5.619 |
| 2 | 15(S)-Hydroxyeicosatrienoic acid | | Fatty Acyls | 7.36_303.2321m/z | | 303.232 | 2.385 | 0.710-0.980 | 0.009 | | 3.450 |
| 3 | 19(S)-HETE | | Fatty Acyls | 1.80_301.2165m/z | | 301.212 | 1.801 | 0.645-0.948 | 0.011 | | 5.428 |
| 4 | 1-Methoxyficifolinol | | Isoflavonoids | 0.59_405.2033m/z | | 405.203 | 0.586 | 0.937-1.000 | 1.370×10^-6^ | | 0.354 |
| 5 | | 21-Deoxycortisol | Steroids and steroid derivatives | 1.06_369.2008m/z | | 369.201 | 1.058 | 0.840-1.000 | 7.140  ×10^-4^ | | 0.523 |
| 6 | | 3-Hexaprenyl-4-hydroxybenzoic acid | Prenol lipids | 0.62_546.4036n | | 585.287 | 0.623 | 0.915-1.000 | | 2.348  ×10^-4^ | 0.314 |
| 7 | | 5-HETE | Fatty Acyls | 1.16_303.2326m/z | | 303.233 | 1.156 | 0.625-0.935 | | 0.011 | 1.601 |
| 8 | | Adrenic acid | Fatty Acyls | 2.98_331.2634m/z | | 331.263 | 2.976 | 0.657-0.953 | | 9.024  ×10^-4^ | 12.561 |
| 9 | | All-trans-heptaprenyl diphosphate | Prenol lipids | | 0.74_655.3895m/z | 655.390 | 0.742 | 0.952-1.000 | | 4.020×10^-5^ | 0.505 |
| 10 | | Avocadyne | Fatty Acyls | | 0.78_329.2318m/z | 329.232 | 0.778 | 0.692-0.975 | | 0.003 | 3.933 |
| 11 | | Bexarotene | Prenol lipids | | 0.68_331.2059m/z | 331.206 | 0.683 | 0.945-1.000 | | 1.910×10^-5^ | 0.462 |
| 12 | | Campesteryl p-coumarate | Steroids and steroid derivatives | | 3.31_564.4395m/z | 564.440 | 3.305 | 0.650-0.950 | | 2.348×10^-4^ | 5.776 |
| 13 | | Ethyl icosapentate | Fatty Acyls | | 2.67_329.2476m/z | 329.248 | 2.668 | 0.647-0.935 | | 0.025 | 5.619 |
| 14 | | Gibberellin A49 | Prenol lipids | | 0.60_345.1331m/z | 345.133 | 0.603 | 0.860-1.000 | | 1.110×10^-5^ | 0.360 |
| 15 | | Glycerol 1-(9Z-octadecenoate) 2-octanoate 3-tetradecanoate | Glycerolipids | | 9.79_692.5967n | 715.586 | 9.791 | 0.655-0.960 | | 0.023 | 0.339 |
| 16 | | Lipoxin A4 | Fatty Acyls | | 6.69_397.2251m/z | 397.225 | 6.687 | 0.700-0.968 | | 0.009 | 1.778 |
| 17 | | Lipoxin B4 | Fatty Acyls | | 6.69_337.2014m/z | 337.201 | 6.687 | 0.775-1.000 | | 0.009 | 1.559 |
| 18 | | LysoPC(16:0) | Glycerophospholipids | | 7.35_480.3088m/z | 480.309 | 7.351 | 0.720-0.975 | | 0.041 | 3.426 |
| 19 | | MG(0:0/15:0/0:0) | Glycerolipids | | 1.23_297.2425m/z | 297.242 | 1.234 | 0.730-0.990 | | 7.159×10^-4^ | 5.303 |
| 20 | | MG(i-22:0/0:0/0:0) | Glycerolipids | | 9.80_437.3635m/z | 437.363 | 9.799 | 0.722-0.978 | | 0.025 | 0.398 |
| 21 | | Porphobilinogen | Organonitrogen compounds | | 0.61_209.0927m/z | 209.093 | 0.614 | 0.800-1.000 | | 2.405×10^-4^ | 0.484 |
| 22 | | Prostaglandin H1 | Fatty Acyls | | 2.45_393.2020m/z | 393.202 | 2.454 | 0.542-0.908 | | 0.028 | 1.810 |
| 23 | | Strigol | Fatty Acyls | | 0.60_331.1152m/z | 331.115 | 0.603 | 0.850-1.000 | | 1.971×10^-4^ | 0.372 |
| 24 | | Testosterone glucuronide | Steroids and steroid derivatives | | 0.59_499.2101m/z | 499.210 | 0.594 | 0.897-1.000 | | 1.158 | 0.319 |
| 25 | | TG(14:0/24:0/16:1) | Glycerolipids | | 7.63_869.8029m/z | 869.803 | 7.630 | 0.755-0.985 | | 0.001 | 6.660 |
| 26 | | TG(14:1/14:1/14:1) | Glycerolipids | | 9.49_716.5981n | 734.632 | 9.490 | 0.740-0.985 | | 0.003 | 0.467 |
| 27 | | TG(14:1/14:1/18:2) | Glycerolipids | | 9.86_793.6345m/z | 793.635 | 9.856 | 0.867-1.000 | | 5.320×10^-5^ | 0.477 |
| 28 | | TG(14:1/16:0/14:1) | Glycerolipids | | 10.13_746.6447n | 769.634 | 10.125 | 0.760-0.985 | | 4.892×10^-4^ | 0.482 |
| 29 | | TG(15:0/14:0/18:3) | Glycerolipids | | 9.86_786.6746n | 809.610 | 9.856 | 0.885-1.000 | | 1.640×10^-5^ | 0.503 |
| 30 | | TG(15:0/14:1/18:1) | Glycerolipids | | 10.13_806.7260m/z | 806.726 | 10.125 | 0.795-0.980 | | 6.602×10^-4^ | 0.503 |
| 31 | | TG(15:0/i-13:0/8:0) | Glycerolipids | | 9.48_661.5416m/z | 661.542 | 9.482 | 0.665-0.970 | | 0.0356 | 0.448 |
| 32 | | TG(18:4/14:1/20:5) | Glycerolipids | | 3.36_860.6706m/z | 860.671 | 3.358 | 0.770-0.998 | | 0.002 | 0.200 |
| 33 | | TG(18:4/20:5/18:4) | Glycerolipids | | 5.71_910.6890m/z | 910.689 | 5.712 | 0.757-0.985 | | 6.432×10^-4^ | 6.046 |
| 34 | | TG(22:4/18:2/22:5) | Glycerolipids | | 10.07_1039.7951m/z | 1039.795 | 10.074 | 0.785-1.000 | | 8.313×10^-4^ | 6.526 |
| 35 | | TG(22:5/20:3n6/22:6) | Glycerolipids | | 3.18_1041.7293m/z | 1041.729 | 3.176 | 0.707-1.000 | | 0.010 | 0.314 |

Compound ID^a^ : The m/z values of the parent molecule fragments RT^b^: Retention time, RT, in min, indicates the time in the liquid chromatograph when the maximum concentration of the component appears after sample entering LC for separation; 95%CI^c^: 95% Confidence Interval; FDR^d^: False discovery Rate; FC^f^: Fold Change.
