# Supplementary material for: LC/MS-based untargeted lipidomics reveals lipid signatures of nonpuerperal mastitis
Source: Lipids Health Dis. 2023 Aug 8;22:122. doi: 10.1186/s12944-023-01887-z (PMC10408177; doi:10.1186/s12944-023-01887-z)
Supplement: Supplementary file 2 — Additional file 2: Supplementary Table S1. Thirty-five differential metabolites between NPM patients and controls [file 12944_2023_1887_MOESM2_ESM.docx]

**Table S2:** **Exact p-values, impact factor values and proportion of altered metabolites for each pathway.**

| **Pathway** | **Match Status** |  | ***P* value** |  | **Impact** |  |
| --- | --- | --- | --- | --- | --- | --- |
| Arachidonic acid metabolism | 12/36 |  | 3.65E-16 | | 0.37419 |  |
| Biosynthesis of unsaturated fatty acids | 4/36 |  | 9.12E-04 |  | 0 |  |
| Glycerophospholipid metabolism | 2/36 |  | 0.076707 |  | 0.12185 |  |
| Glycosylphosphatidylinositol (GPI)-anchor biosynthesis | 1/14 |  | 0.16689 |  | 0.00399 |  |
| Steroid hormone biosynthesis | 2/85 |  | 0.30081 |  | 0.02345 |  |
| Porphyrin and chlorophyll metabolism | 1/30 |  | 0.3252 |  | 0.05288 |  |

Match Status：The preceding number indicates the number of metabolites screened in the pathway. The second number indicates the

number of all metabolites contained in the pathway. *p* values from the pathway enrichment analysis and pathway impact values from

the pathway topology analysis.
