# Supplementary material for: LC/MS-based untargeted lipidomics reveals lipid signatures of nonpuerperal mastitis
Source: Lipids Health Dis. 2023 Aug 8;22:122. doi: 10.1186/s12944-023-01887-z (PMC10408177; doi:10.1186/s12944-023-01887-z)
Supplement: Supplementary file 3 — Additional file 3: Supplementary Table S2. Exact p values, impact factor values and proportion of altered lipids for each pathway [file 12944_2023_1887_MOESM3_ESM.docx]

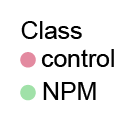


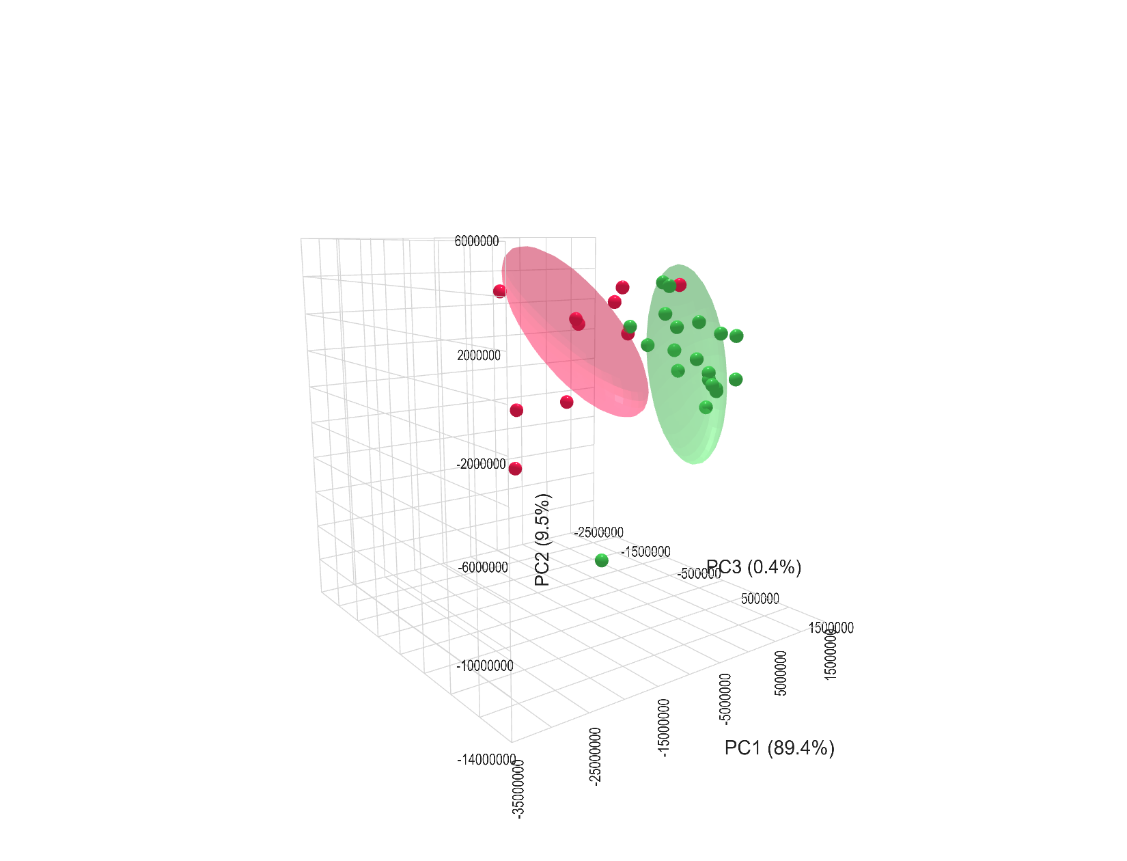


Fig S2. PCA score plots of the 35 identified lipids in 3D mode. We conducted a comprehensive analysis of 35 potential lipid biomarkers using scoring, VIP, and pathway analysis. Additionally, we employed the PCA dimensionality reduction statistical method to assess the ability of the identified lipids to effectively differentiate between control and the NPM.
