# Supplementary material for: LC/MS-based untargeted lipidomics reveals lipid signatures of nonpuerperal mastitis
Source: Lipids Health Dis. 2023 Aug 8;22:122. doi: 10.1186/s12944-023-01887-z (PMC10408177; doi:10.1186/s12944-023-01887-z)
Supplement: Supplementary file 4 — Additional file 4: Fig S2. PCA score plots of the 35 identified lipids in 3D mode [file 12944_2023_1887_MOESM4_ESM.pdf]

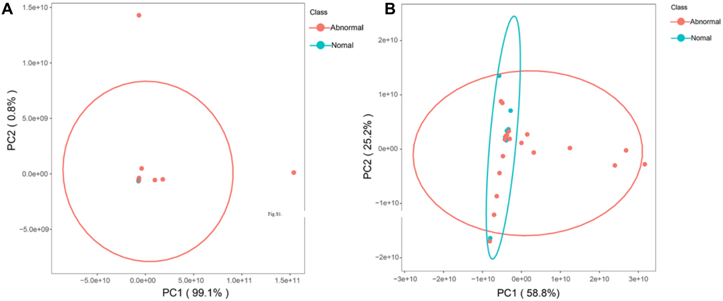

Fig S1. PCA score plots of the abnormal HDL and normal HDL (NPM:n=20, Control:n=7).A:Negative mode; B: Positive mode.
